# Supplementary material for: Comparison of Coupled Electrochemical and Thermal Modelling Strategies of 18650 Li-Ion Batteries in Finite Element Analysis—A Review
Source: Materials (Basel). 2023 Dec 12;16(24):7613. doi: 10.3390/ma16247613 (PMC10744660; doi:10.3390/ma16247613)
Supplement: Supplementary file 1 [file materials-16-07613-s001.zip › Figure S3.pptx]

## Slide 1
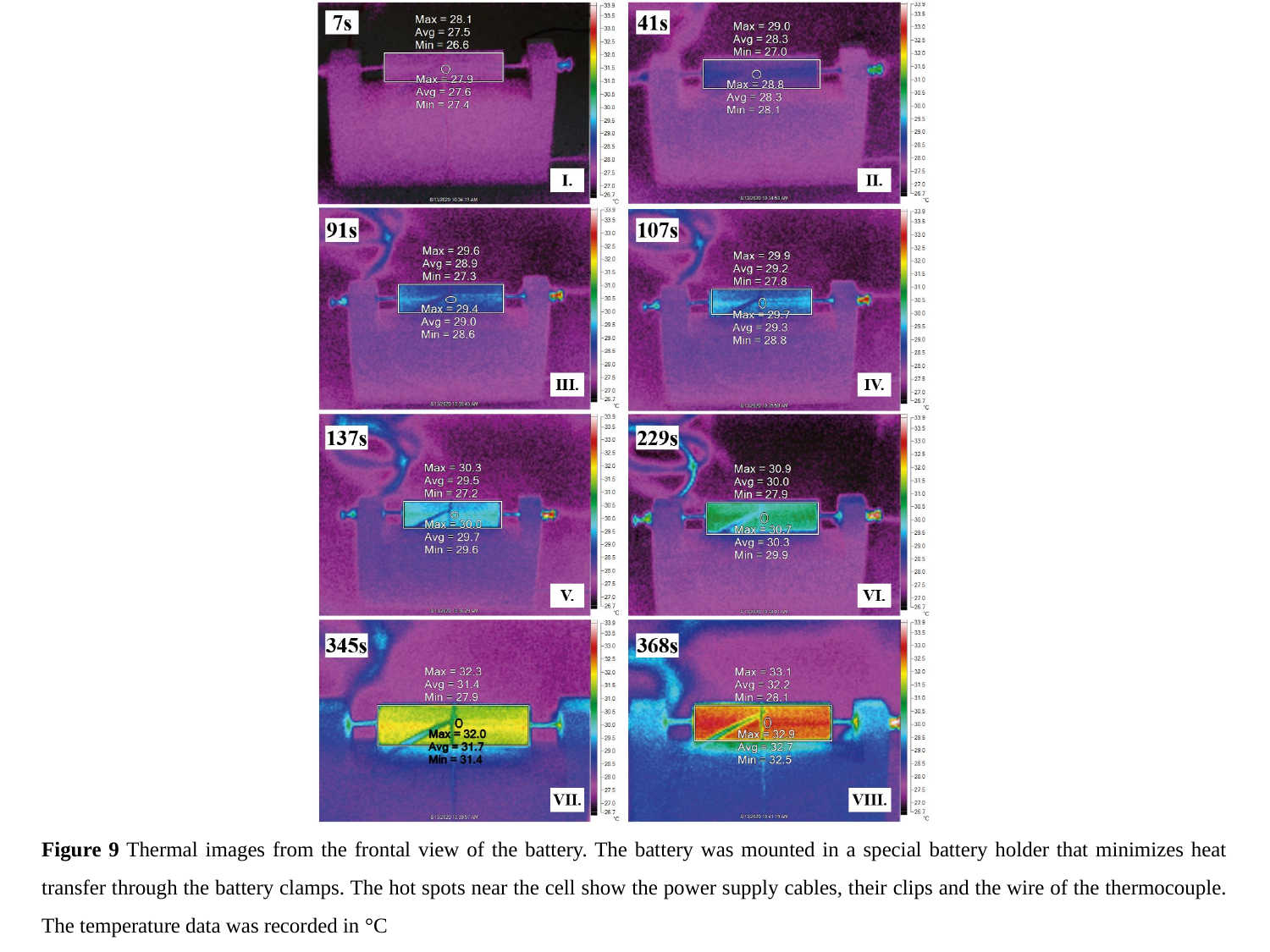

Figure 9 Thermal images from the frontal view of the battery. The battery was mounted in a special battery holder that minimizes heat transfer through the battery clamps. The hot spots near the cell show the power supply cables, their clips and the wire of the thermocouple. The temperature data was recorded in °C
