# Supplementary material for: Comparison of Coupled Electrochemical and Thermal Modelling Strategies of 18650 Li-Ion Batteries in Finite Element Analysis—A Review
Source: Materials (Basel). 2023 Dec 12;16(24):7613. doi: 10.3390/ma16247613 (PMC10744660; doi:10.3390/ma16247613)
Supplement: Supplementary file 1 [file materials-16-07613-s001.zip › Figure S4.pptx]

## Slide 1
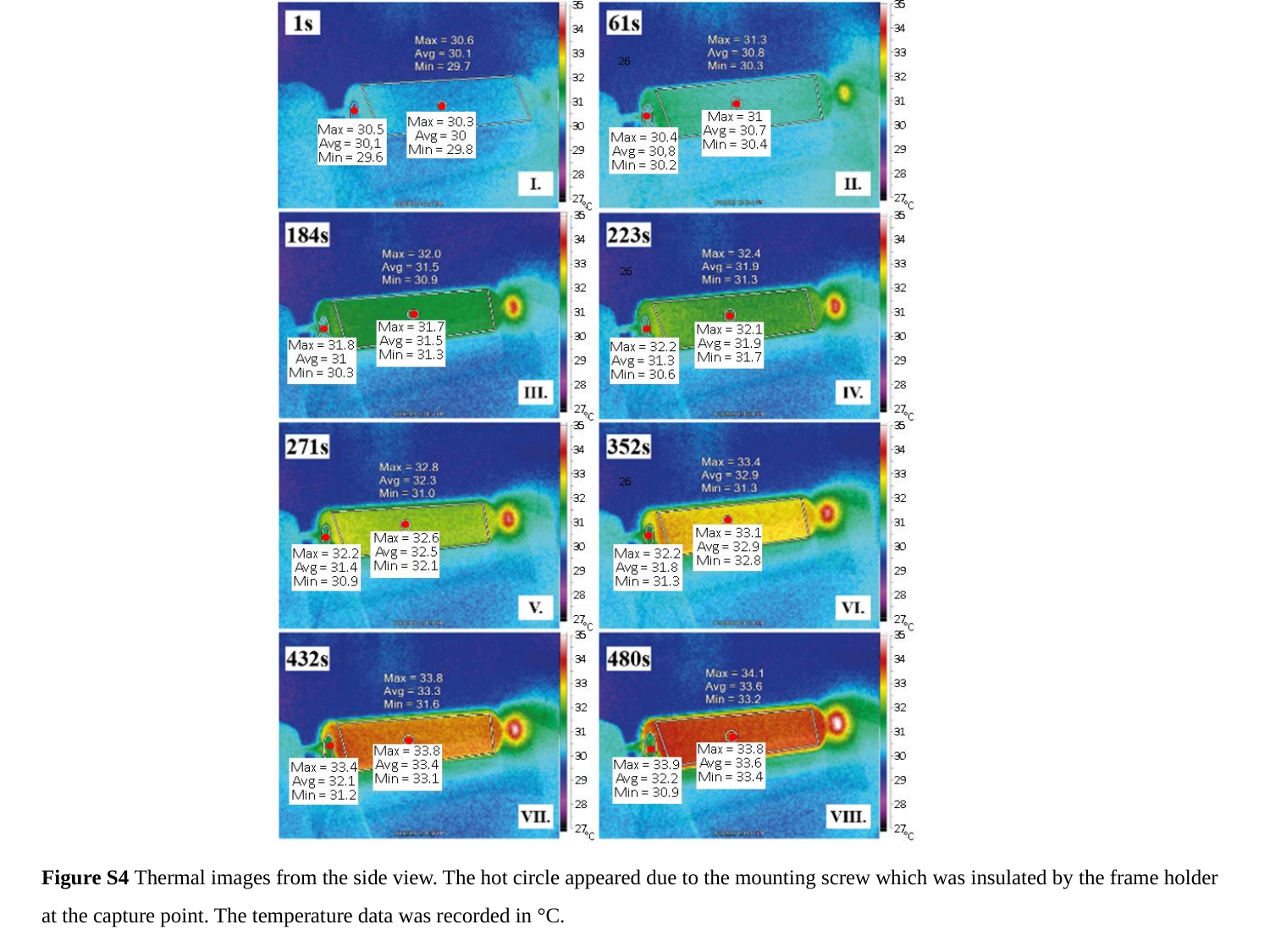

Figure S4 Thermal images from the side view. The hot circle appeared due to the mounting screw which was insulated by the frame holder at the capture point. The temperature data was recorded in °C.
