# Supplementary figures and images for: Comparison of Coupled Electrochemical and Thermal Modelling Strategies of 18650 Li-Ion Batteries in Finite Element Analysis—A Review
Source: Materials (Basel). 2023 Dec 12;16(24):7613. doi: 10.3390/ma16247613 (PMC10744660; doi:10.3390/ma16247613)

## Slide 1
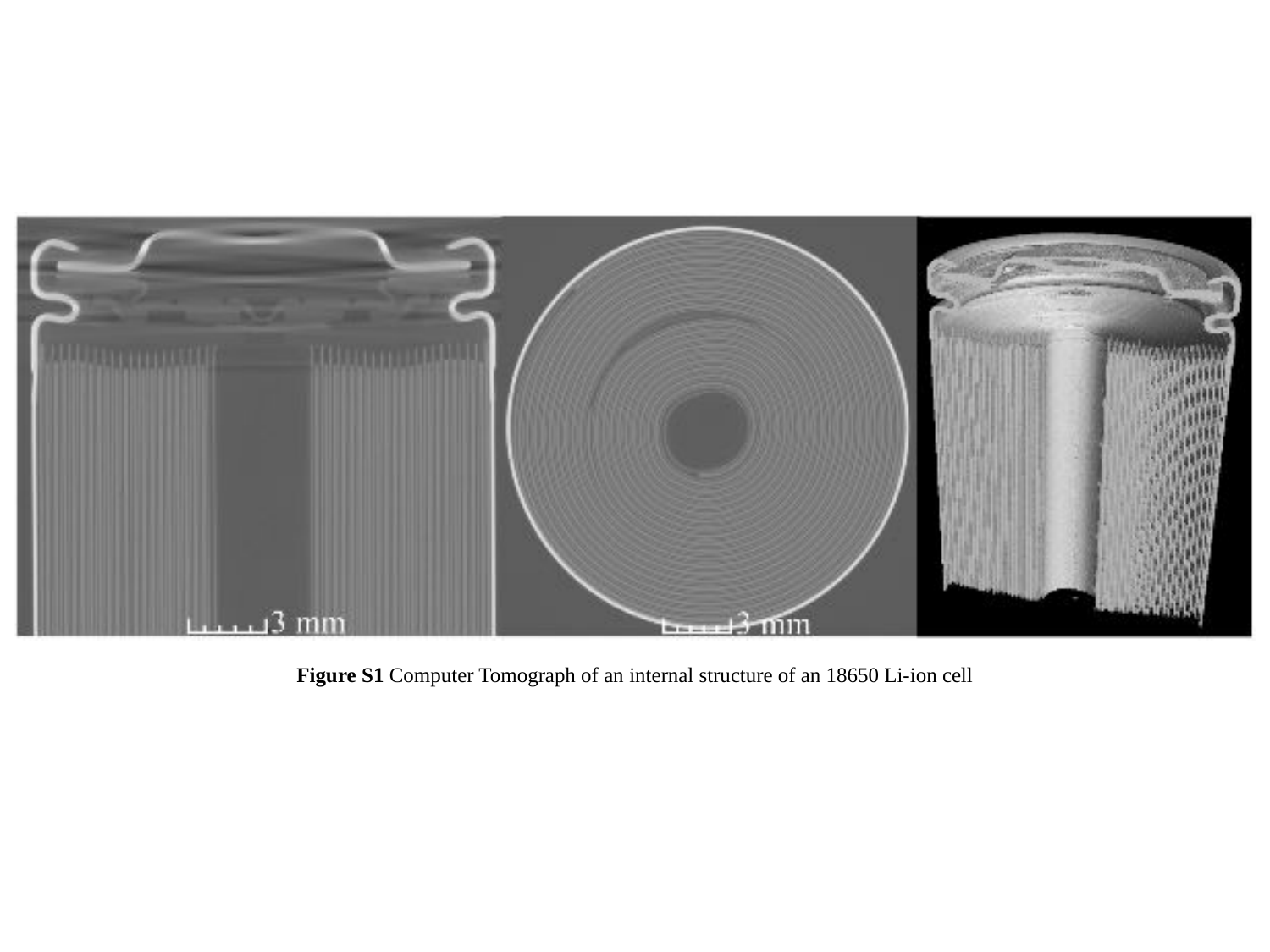

Figure S1 Computer Tomograph of an internal structure of an 18650 Li-ion cell

Supplement: Supplementary file 1 [file materials-16-07613-s001.zip › Figure S1.pptx]

## Slide 1
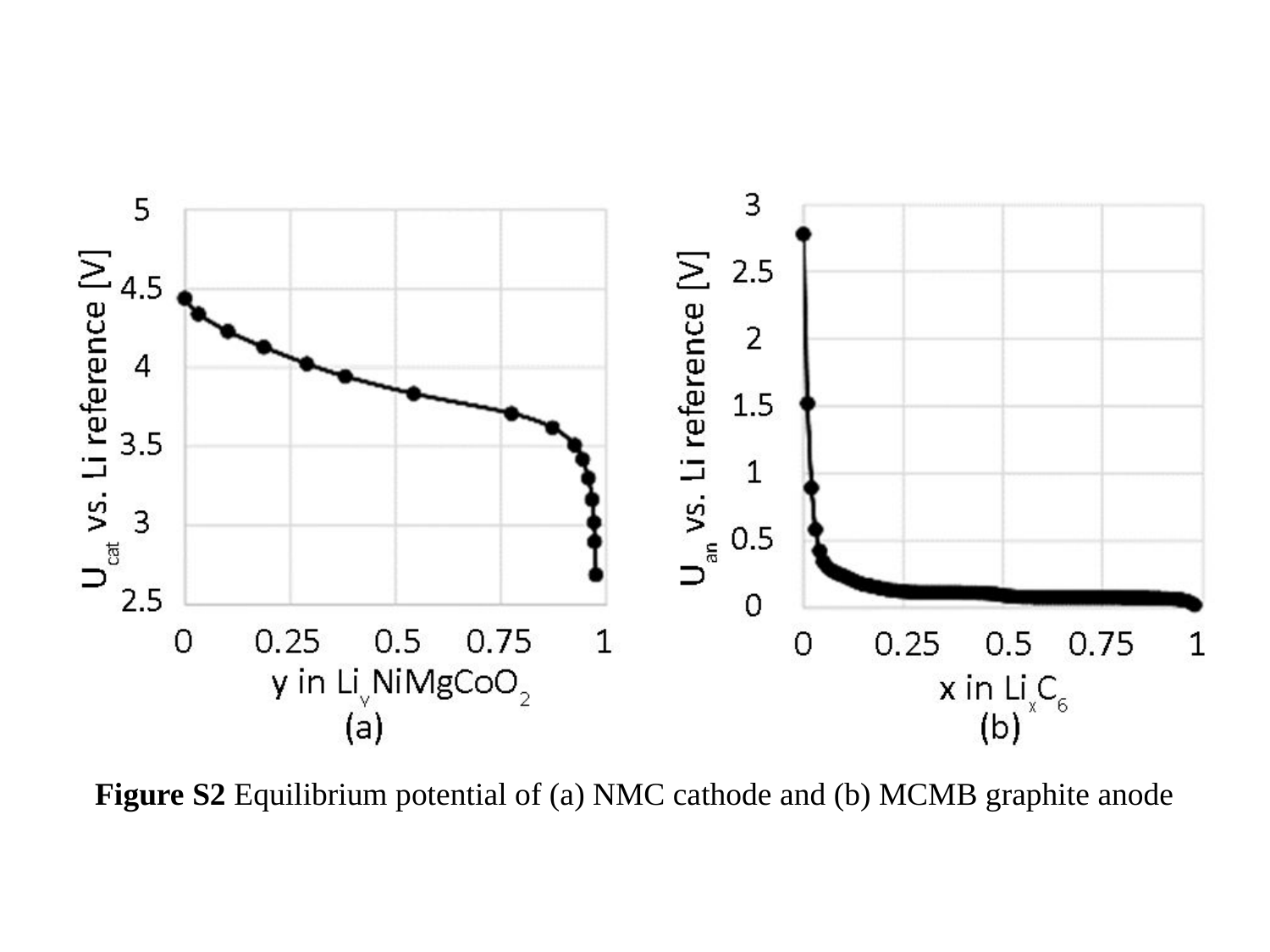

Figure S2 Equilibrium potential of (a) NMC cathode and (b) MCMB graphite anode

Supplement: Supplementary file 1 [file materials-16-07613-s001.zip › Figure S2.pptx]

## Slide 1
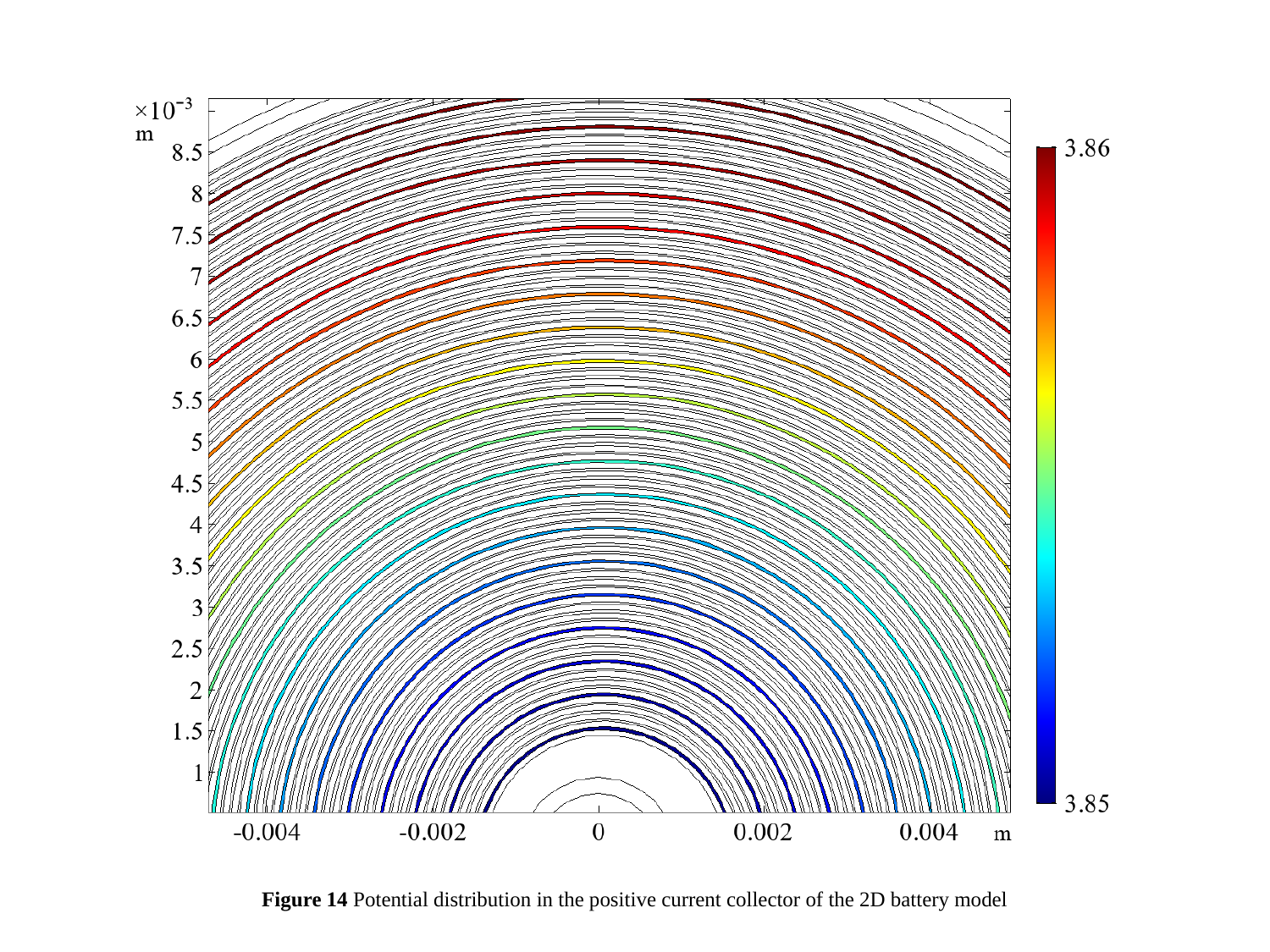

Figure 14 Potential distribution in the positive current collector of the 2D battery model

Supplement: Supplementary file 1 [file materials-16-07613-s001.zip › Figure S5.pptx]

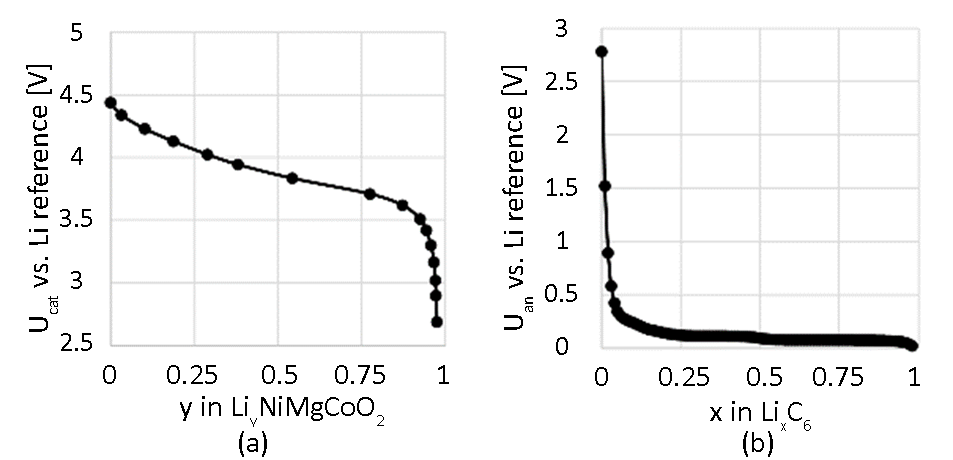

Supplement: Supplementary file 1 [file materials-16-07613-s001.zip › FigureS2.png]

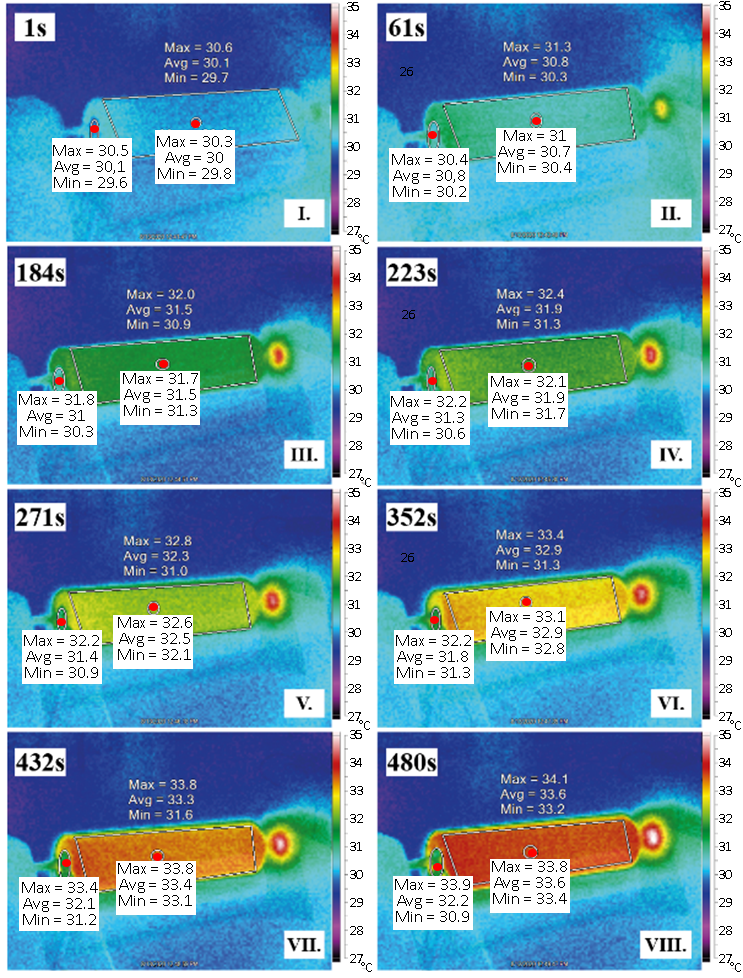

Supplement: Supplementary file 1 [file materials-16-07613-s001.zip › FigureS4.png]
